# Spanish Transcultural Adaptation of the Activity Card Sort

**DOI:** 10.1155/2019/4175184

**Published:** 2019-09-10

**Authors:** Cristina Alegre-Muelas, Jorge Alegre-Ayala, Elisabet Huertas-Hoyas, MªRosa Martínez-Piédrola, Jorge Pérez-Corrales, Nuria Máximo-Bocanegra, Carlos Sánchez-Camarero, Marta Pérez-de-Heredia-Torres

**Affiliations:** ^1^Occupational Therapy Department, Instituto San José, Cart. Barrio de la Fortuna, 0, CP.28022 Madrid, Spain; ^2^Occupational Therapy Department, Centro FOREN, Ronda de Poniente, 12, CP.28760 Tres Cantos, Spain; ^3^Rehabilitation and Physical Medicine Department, Rey Juan Carlos University, Avenida de Atenas s/n, CP.28922 Alcorcón, Madrid, Spain

## Abstract

The Activity Card Sort (ACS) measures the level of participation, as perceived by each person which, unlike other scales, makes it both personal and significant. However, there is a limitation to applying the ACS to Spanish older adults as it is restricted to culturally relevant activities solely in the United States. Therefore, the aim of this study was to select activity items that reflected Spanish older adults' lifestyles in order to develop the Activity Card Sort-Spain Version (ACS-SP). Frequently, activities performed in Spain (*n* = 103) were listed in an initial draft. The Likert scale was administrated to a large group of Spanish nationals over the age of 60 years (*n* = 98) to establish which type of activities will be eventually included in the Spanish version. The final version was drawn up comprising 79 activities distributed between four performance areas. In addition, other activities that were not previously included by other assessment tools were considered and have been listed in this review, such as taking a nap, going out for a drink or “tapas,” or searching for a job. The gradual adaptation to ACS for Spaniards will make it possible to measure the level of an individual's participation within a community. However, further work on psychometric properties is needed.

## 1. Introduction

Participation entails a connection between the person, their specific context, and the tasks performed. Thus, participation occurs when a person performs, or wishes to perform, an activity, has the opportunity to undertake the same, and has overcome any challenges that might limit their engagement in the activity at the preferred location [1].

The importance of community participation is highlighted as being a key as an indication that the rehabilitation process has been a success [2]. To be able to participate once again in daily activities is one of the most valuable outcomes for people with a disability, as well as for their family members and the society as a whole [3]. Also, for noninstitutionalized persons who are independent enough to carry out activities of daily living, their participation in recreational activities, as part of a community, may delay the onset of the dependence associated with ageing [4].

At present, despite the importance of participation, problems with the definition continue to exist and are often confused with related concepts, such as the health-related quality of life or community integration [5].

The Activity Card Sort (ACS) is a tool that was developed by occupational therapists in the United States [6]. This instrument measures the level of participation, as perceived by the person screened, via the use of picture cards depicting daily activities. These activities are categorized into four areas: instrumental activities, low physical demand leisure activities, high physical demand leisure activities, and socioeducational activities. This scale covers eight of the nine participation domains listed by the International Classification of Functioning, Disability and Health (ICF) [7, 8]. The ACS presents appropriate psychometric characteristics, with a high level of internal consistency (>0.85) and construct validity (>0.60) and a good level of test-retest reliability (0.88-0.95) [9].

Although originally configured as a scale for people with Alzheimer's disease, this scale has been used among other populations, such as on individuals with Parkinson's disease [10, 11] and brain damage [12, 13] or older people [14, 15]. Several versions are available according to the assessment goal (institutional version, people living in their own home, and those recovering after the development of a rehabilitation intervention). The ACS has been adapted to different countries and cultures, such as Japan (ACS-JPN) [16], Israel (ACS-Israeli version) [17], Australia (ACS-Aus) [18], Hong Kong (ACS-HK) [19], Puerto Rico (PR-ACS) [20], United Kingdom (ACS-UK) [21], Holland (ACS-NL) [22], and Arab Heritage (A-ACS) [23]. A systematic approach has been applied to develop culturally relevant versions worldwide although a gap exists in the Spanish population.

There are few scales validated for Spanish speakers that enable the assessment of participation. Some examples of available scales are the Community and Socio-Political Participation Scale (SCAP) [24], the Children's Assessment of Participation and Enjoyment (CAPE) [25], the Social Functioning Scale (SFS) [26], and the Leisure Assessment Index (LAI) [27]. However, the main aim of these assessments is not to analyze participation in the community for adults living in their own home, but rather they have been designed for specific populations, such as adults with intellectual disabilities or mental illness. In addition, they examine fewer dimensions of participation collected by the ICF and analyze a smaller number of activities than the ACS scale [7]. However, none of these present such a wide number of items for the assessment of community participation, nor have they been used for assessing participation from such a wide spectrum of illnesses, when compared to the ACS scale [24, 25, 27] (Vázquez-Morejón and Jiménez Ga-Bóveda 2012). Considering its previous use among different populations and the level of reliability and validity, the ACS is considered a highly valuable tool for examining the level of community participation among community-dwelling adults in Spain. Despite the existence of common activities between both the American and the native Spanish populations, an adaptation process for this scale is needed, together with changes in content. This means that activities that are not considered to be common in Spain should be removed, whereas other more common activities should be introduced as, for example, is done in Japan, Great Britain, Australia, and Hong Kong [16, 18, 19] (Laver-Fawcett et al. 2013). This will enable the development of an effective tool for monitoring the level of participation among Spanish community-dwelling adults.

The aim of this study was to select activity items reflecting Spanish older adults' lifestyles and develop a Spanish version of the ACS scale (ACS-SP).

## 2. Materials and Methods

### 2.1. Participants

**Figure 1:**
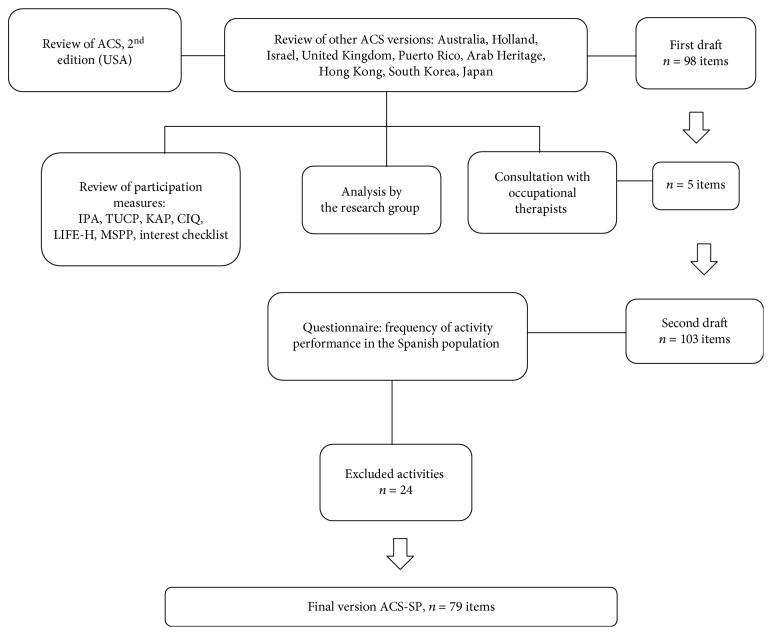
Adaptation process of the ACS scale to the Spanish population.

The study participants came from different regions in Spain and were well known by the researchers' families and/or work circle. The participants who responded to the questionnaire, regarding the frequency of their performance of daily living activities in Spain, were recruited according to the following inclusion criteria: people living in the community and who were able to comprehend and communicate in Spanish, people who had retired from their full-time jobs or were homemakers from the outset, and those aged 60 years or older.  Figure 1 summarizes the process of adaptation followed.

### 2.2. Generation of Items for the Scale

In order to develop the Spanish adaptation of the ACS scale, we first analyzed the second edition of the ACS [28]. Thereafter, we selected the daily living activities on this scale that were also considered common in Spain, discarding those that were less common, or which could be included in self-care activities (such as resting), at the discretion of the research team. The research group analyzed whether the activities within this scale were frequent in Spain and whether they were part of the Spanish culture, based on the second edition of the scale. Subsequently, the research group analyzed the different versions of the ACS to detect other daily living activities that were not found in the original ACS version in order to include other activities that could be considered common for people in Spain.

Thereafter, the different adaptations of the scale performed in other countries were reviewed for the purpose of selecting activities considered common in Spain. With this aim, the following ACS versions were consulted: the ACS-Israeli version [17], the ACS-Aus [18], the PR-ACS [20], the ACS-UK [21], the ACS-NL [22], and the A-ACS [23]. Also, other community participation measures and some occupational therapy evaluation instruments were evaluated to extract more activities that had not been collected by the ACS versions. These included the Impact on Participation and Autonomy (IPA) [29], the Temple University Community Participation (TUCP) [30], the Assessment of Life Habits (LIFE-H) [31], the Community Integration Questionnaire (CIQ) [32], the Keele Assessment of Participation (KAP) [33], the Maastricht Social Participation Profile (MSSP) [34], and the Interest Check List, all of which are considered additional useful instruments for occupational therapists to learn about additional significant activities [35]. This analysis produced the initial draft of activities that are currently practiced in Spain.

After this initial analysis, a group of occupational therapists (*n* = 12), with an average experience of 10.5 years (3.92) (min–max, 3-15), working in different fields, analyzed whether the activities appearing in the initial draft were frequent in Spain and whether some items were less common.

After gathering this information, the research team established an initial list of items. This list was administered as a questionnaire to people aged 60 years and older, without health problems, and from various regions in Spanish. Following the examples of the ACS-Aus, ACS-UK, and the A-ACS [18, 21, 23], a Likert scale was administered in which the assessed person was asked to reflect on the frequency of the specified activities which were performed in Spain. For each activity, five optional responses were available (“0” = nobody performs that activity, “1” = few people perform that activity, “2” = some people perform that activity, “3” = many people perform that activity, and “4” = the vast majority of people perform that activity). For each item, we calculated the mean and its standard deviation.

The research group decided that the final version should only include those activities that presented scores equal to or greater than two, as is done in the ACS-UK version [21], discarding all those with lower scores. Finally, the activities included in the Spanish version of the ACS were separated into three distinct dimensions: instrumental, leisure, and social participation activities and productivity and education. To prepare the images used on the Spanish version cards of the ACS scale, Spanish nationals of between 60 and 75 years old were photographed.

### 2.3. Adaptation Process

Permission was first obtained from the authors of the test for the translation and validation of the same among the Spanish population. We then obtained authorization from the Rey Juan Carlos University Ethics Committee.

### 2.4. Statistical Analysis

The calculation of the mean scores was performed using the IBM SPSS Statistics 22.0 for Windows (IBM Corporation, Armonk, NY, USA) statistical program.

## 3. Results

Based on the analysis of the different versions of the ACS scale, the first draft produced a list of activities (*n* = 98). After the review of the participation outcome measures, via the consensus of the research group, and after a consultation with other occupational therapists, additional items were added (*n* = 5). This resulted in the Likert questionnaire (*n* = 103) which was then presented to the study participants.

These participants came from different regions in Spain: Madrid, Castilla-La Mancha, Castilla y León, Extremadura, Canarias, Cataluña, and Aragón (*n* = 98). However, the majority were from Madrid (*n* = 55), rather than from other Spanish regions (*n* = 43) (including rural areas, towns, and metropolitan cities). The mean age of the sample was 63.59 ± 4.9 (min–max, 60-80). Of the total participants, 66 (66.7%) were women and 32 (23.3%) were men. The remaining characteristics of the sample are displayed in Table 1.

Once the questionnaires had been administered to the participants, the final version of the ACS-SP was drafted by including all items with scores equal to or greater than two (*n* = 79).  Table 2 displays these on the scale ordered by their mean score. The most frequent activity was watching television (3.77 ± 0.54), whereas taking care of/watering plants was the least frequent activity cited (2.01 ± 0.72). The activities excluded from the final version (*n* = 24) are displayed in Table 2.

The activities (*n* = 79) were categorized into the following four areas: instrumental activities (*n* = 26), leisure activities (*n* = 23), social participation activities (*n* = 27), and productivity and education activities (*n* = 3), as displayed in Table 3.

The items included in the final version of the scale primarily arose from the second edition of the ACS (*n* = 61). Those remaining were extracted from ACS-Aus (*n* = 2), A-ACS (*n* = 2), ACS-UK (*n* = 7), and ACS-NL (*n* = 3) and a consultation with occupational therapists (*n* = 1) or were added by the group of researchers (*n* = 3).

With regard to the changes made to the sample Spanish population, three new activities were included: taking a nap, going out for a drink or tapas, and searching for a job. Although the meaning of these words may appear similar to the original ACS (resting, clubbing, or entertaining), they differ due to the cultural difference, resulting in a different meaning. For example, taking a nap in Spain is not just resting; it is a short sleep period taken between productive activities, after lunch (midday).

## 4. Discussion

The process of crosscultural adaptation of the ACS to the Spanish population resulted in a list of 79 activities, divided into four distinct areas: instrumental activities, leisure activities, social participation activities, and productivity and education.

The final list was developed after both reviewing the available international versions of the ACS and considering the contributions made by different occupational therapists, as well as after analyzing the responses provided by a group of people over the age of 55 regarding the frequency of the selected activities they considered being performed in Spain. This process of item generation based on population surveys was also used for the development of the ACS-Aus, ACS-UK, and A-ACS versions of the scale [18, 21, 23]. Unlike our study, none of the versions of the ACS had previously revised other measurements of participation in the community in order to generate items. Only one scale (ACS-UK, Laver-Fawcett et al. 2013) consulted other occupational therapists (aside from the group of researchers) in order to review and generate more items that could be considered common among the Spanish population.

The total number of activities included in the Spanish version is similar to that in ACS-NL (*n* = 79), although greater than the number of items used in ACS-HK (*n* = 65) [19]. However, the final Spanish version presents a smaller number of items than the original ACS version (*n* = 82), the second edition (*n* = 89), and the ACS-Israeli version (*n* = 87), ACS-Aus (*n* = 82), PR-ACS (*n* = 82), ACS-UK (*n* = 91), and A-ACS (*n* = 88). An example of a participation measurement that evaluates Spanish seniors is the SCAP [24]. This scale focuses on a few sections of both community participation and specific aspects of sociopolitical participation, such as participating in associations and NGOs, volunteering, attending debates, attending social events within their community, voting, being part of political parties, or participating in demonstrations. Another Spanish measurement that has assessed various dimensions of participation is the SFS [26], although its aim is to evaluate the level of social participation in people with schizophrenia. The dimensions evaluated are social activities, leisure and employment, and instrumental and self-care activities. It also includes an item which analyzes social isolation. The number of items that the scale includes is lower than that of the instrument presented in this study. In addition, this scale analyzes the frequency that some of the activities take place within a certain time interval or the level of assistance that may be required for certain activities. Finally, another example of a Spanish scale that examines participation focuses only on the leisure section and is designed for individuals with intellectual disabilities [27].

Regarding the number of items that coincide with other versions, it is worth noting that the final ACS-SP version includes 61 activities listed in the 2^nd^ edition of the ACS. In the case of ACS-Aus, both versions include 65 similar activities while, in the case of the version for adults between 18 and 64 years, 72 activities coincide [36]. Both the ACS-UK and our version present 66 similar activities, sharing 63 items with the A-ACS, 59 items with the ACS-NL, and 57 activities with the ACS-Israeli version.

Compared to the second edition of the ACS, 30 activities from the former version are unlisted and 21 new activities have been included. The activities excluded from the Spanish version are performing DIY, resting, cooking as a hobby, sewing, doing handicrafts, doing macramé/petit poi embroidery, collecting, doing puzzles, playing musical instruments, reading the bible, singing in a choir, writing, writing a letter, bird watching, going to the casino, going to bingo, sitting and thinking, doing carpentry activities, going bowling, playing golf, garden games, sailing, fishing, going to church, spending time with a spouse, volunteering, going to a club, and going on a picnic. Shopping at a supermarket and buying food were combined as a “going shopping” activity. Similarly, travelling locally and travelling to other countries were combined as “travelling,” and swimming was categorized as “going to the pool.”

Twenty new activities have been included into the ACS-SP, which are not included in the second edition of the ACS American version: taking care of young children, taking care of people who are ill, using the computer, packing bags, using public transport, planning a trip, performing administrative tasks, organizing cupboards and clothes, internet surfing, job searching, betting/gambling, going to the beach, going to the gym, taking a nap, taking children to different extracurricular activities, going out for a drink, going to a funeral, going to exhibitions, and voting.

Regarding the differences between the ACS-SP and the remaining versions, this version contains three items that do not appear in the rest of the scales, namely, going out for a drink, taking a nap, and searching for a job. Going out for a drink and taking a nap are very typical activities among the Spanish population which are not necessarily performed in other countries. Taking a nap is not just resting; it is a short sleep period taken between productive activities, after lunch (midday), and sometimes can extend to the period of one hour. Spanish residents are accustomed to taking a nap, and this is considered a well-established Spanish custom.

Regarding searching for a job, the occupational therapists consulted felt that it was advisable to include as it was also commonplace in Spain; thus, it was included as being both a productive activity and an educational activity and in different sections, as the Association of American Occupational Therapy considers that this can be categorized as two different activities [37].

Furthermore, our version was the only one, together with the ACS-UK [21], to include voting and going to the dentist.

On the other hand, the final version of the scale does not include activities, such as going to a religious center or reading religious material, both of which were included in the other available versions [17, 18, 20–23, 36]. Similarly, within the items listed on this scale, we did not consider leisure activities, such as doing handicrafts, sewing, doing puzzles, or collecting, neither social activities, such as volunteering, which do appear in the original ACS version, the second ACS edition, the ACS-Israel version, ACS-Aus, ACS-UK, or ACS-NL [6, 17, 18, 21, 22, 28]. The absence of activities related to religion and volunteering in the final version could be explained due to the low participation of the Spanish population in these types of activities and the decrease in the number of active participants within these communities/practitioners [38, 39].

Our scale was divided into four dimensions: instrumental activities, leisure activities, social participation, and productivity and education. This is a different criterion compared to other versions as we did not consider dividing leisure activities according to their physical demand; rather, the corresponding activities were classified as being “leisure” due to the inclusion of leisurely activities.

### 4.1. Limitations

This work presents several limitations. In the first place, it would be advisable to have included younger age groups in the population sample for comparative purposes to obtain the most representative activities in a larger sample. Secondly, our study did not analyze the results considering the origin of the participants as in whether these came from urban and rural areas; therefore, it is possible that some activities may not be so representative in certain contexts.

## 5. Conclusion

This paper has detailed the process for adapting the ACS scale to the Spanish population by generating a new scale entitled ACS-SP. This scale may be used across a wide variety of settings and situations. The majority of the items on this scale have already been used in the different versions of the ACS that have been developed in other countries. However, after a process of analysis and a survey of a small sample of the Spanish population, the most frequent activities were included, some of which are unique when compared to other versions. The ACS-SP will serve as a descriptive tool for assessing community participation among Spanish community-dwelling adults and may be a useful outcome measure for determining the effects of rehabilitation interventions on participation and for establishing tailored occupation-focused goals.

The adaptation to ACS for the Spanish native population will make it possible to measure the level of an individual's participation in a community, in this case with elderly people living at their place of residence, to whom a broad and comprehensive set of performance areas will be addressed.

## Figures and Tables

**Table 1:** Characteristics of the study participants (*n* = 98).

Variable		Madrid (*n* = 55)	Other regions (*n* = 43)	Total (*n* = 98)
Sex	Female, *n* (%)	37 (67.3)	29 (67.4)	66 (66.7)
	Male, *n* (%)	18 (32.7)	14 (32.6)	32 (32.3)

Age, mean ± SD (min–max)		64.13 ± 5.51 (60-80)	62.91 ± 3.95 (60-72)	63.59 ± 4.9 (60-80)

Education	Basic, *n* (%)	12 (21.8)	10 (23.3)	22 (22.2)
	Secondary, *n* (%)	23 (41.8)	12 (27.9)	35 (35.4)
	University, *n* (%)	20 (36.4)	21 (48.8)	41 (41.4)

Marital status	Single, *n* (%)	3 (5.5)	2 (4.7)	5 (5.1)
	Widow/er, *n* (%)	13 (2.3)	3 (7)	16 (16.2)
	Married, *n* (%)	37 (67.3)	36 (83.7)	73 (73.7)
	Divorced, *n* (%)	2 (3.6)	2 (4.7)	4 (4)

**Table 2:** Activities included and excluded in the final version of the ACS-SP ordered according to the frequency estimated by participants (*n* = 79 and *n* = 24, respectively).

Activities by ranking number	Mean (SD)
(1) Watching television	3.77 (0.54)
(2) Shopping (supermarket, shop)	3.73 (0.44)
(3) Talking on the phone	3.62 (0.73)
(4) Going to shopping centers	3.40 (0.84)
(5) Going to a doctor's appointments	3.32 (0.70)
(6) Taking out the rubbish	3.31 (0.92)
(7) Paying bills	3.24 (0.85)
(8) Family events	3.21 (0.83)
(9) Voting	3.14 (0.66)
(10) Surfing on the internet	3.13 (0.82)
(11) Listening to music	3.12 (0.70)
(12) Driving	3.09 (0.75)
(13) Filling up on petrol	3.09 (0.76)
(14) Working	3.08 (0.79)
(15) Cooking	3.05 (0.68)
(16) Having a coffee	3.04 (0.73)
(17) Visiting family/friends (ill)	3.04 (0.74)
(18) Listening to the radio	3.04 (0.91)
(19) Visiting friends	3.03 (0.81)
(20) Going out for a drink	3.03 (0.84)
(21) Going to the beach	3.01 (0.72)
(22) Using the computer	3.00 (0.73)
(23) Using social media	3.00 (0.84)
(24) Spending time with friends	2.99 (0.89)
(25) Washing dishes	2.96 (0.80)
(26) Using public transport	2.95 (0.66)
(27) Housekeeping (ironing, cleaning, making beds)	2.86 (0.79)
(28) Going shopping (leisure)	2.85 (0.84)
(29) Walking	2.83 (0.77)
(30) Taking a nap	2.83 (0.81)
(31) Going to the hairdresser	2.81 (0.88)
(32) Washing clothes	2.81 (1.10)
(33) Travelling	2.80 (0.69)
(34) Going to funerals	2.78 (0.90)
(35) Betting, gambling	2.78 (0.91)
(36) Taking care of young children	2.76 (0.70)
(37) Going to the cinema	2.76 (0.78)
(38) Taking the car to the mechanic	2.75 (0.85)
(39) Reading	2.70 (0.73)
(40) Taking care of pets	2.67 (0.81)
(41) Going to parties	2.66 (0.74)
(42) Packing bags	2.65 (0.76)
(43) Going to restaurants	2.65 (0.84)
(44) Taking children to extracurricular activities	2.62 (0.85)
(45) Going to the park	2.57 (0.82)
(46) Playing videogames	2.56 (0.87)
(47) Exercising	2.53 (0.66)
(48) Taking photos	2.51 (0.82)
(49) Studying	2.49 (0.79)
(50) Reading newspapers	2.49 (0.85)
(51) Going to see sports games (sports stadiums)	2.48 (0.92)
(52) Handling finances (investments, going to the bank)	2.42 (0.91)
(53) Sorting out cupboards, clothes	2.41 (0.92)
(54) Planning a trip	2.40 (0.81)
(55) Going to the swimming pool	2.39 (0.83)
(56) Going to the gym	2.38 (0.78)
(57) Going to the dentist	2.37 (0.73)
(58) Job searching	2.37 (0.81)
(59) Reading stories to children	2.36 (0.82)
(60) Taking care of ill people	2.31 (0.83)
(61) Doing administrative tasks	2.30 (0.78)
(62) Going to concerts	2.29 (0.76)
(63) Doing team sports	2.21 (0.64)
(64) Visiting exhibitions	2.21 (0.80)
(65) Riding a bicycle	2.20 (0.78)
(66) Going to the theatre	2.18 (0.72)
(67) Going running	2.17 (0.64)
(68) Going dancing	2.16 (0.71)
(69) Having a party at home	2.15 (0.78)
(70) Doing crossword puzzles, pastimes	2.14 (0.80)
(71) Playing card games	2.13 (0.67)
(72) Hiking	2.13 (0.82)
(73) Home decorating	2.11 (0.76)
(74) Visiting museums	2.09 (0.79)
(75) Doing yoga, Pilates, tai chi	2.07 (0.71)
(76) Going camping	2.07 (0.82)
(77) Board games	2.06 (0.71)
(78) Playing tennis, paddle tennis	2.01 (0.69)
(79) Taking care of/watering plants	2.01 (0.72)
Activities excluded from the final version according to the participants (*n* = 24)	
(80) Collecting	1.99 (0.81)
(81) DIY	1.97 (0.69)
(82) Belonging to associations	1.97 (0.74)
(83) Cooking as a hobby	1.96 (0.67)
(84) Going to church	1.88 (0.74)
(85) Going to the post office	1.88 (0.82)
(86) Going to bull fighting events	1.86 (0.66)
(87) Going to bingo	1.86 (0.84)
(88) Painting	1.85 (0.73)
(89) Going to the library	1.84 (0.76)
(90) Changing the butane cylinder	1.83 (0.86)
(91) Sewing	1.83 (0.92)
(92) Playing a musical instrument	1.82 (0.80)
(93) Doing handicrafts	1.80 (0.67)
(94) Volunteering	1.71 (0.80)
(95) Doing puzzles	1.64 (0.65)
(96) Going to the casino	1.55 (0.64)
(97) Fishing	1.55 (0.68)
(98) Singing in a choir	1.51 (0.68)
(99) Carpentry activities	1.47 (0.57)
(100) Bowling	1.47 (0.60)
(101) Reading the bible	1.47 (0.71)
(102) Knitting/macramé	1.45 (0.66)
(103) Writing	1.36 (0.53)

**Table 3:** Distribution of the activities included in the final version of the ACS-SP by areas.

Instrumental (*n* = 26)	Leisure (*n* = 23)	Social participation (*n* = 27)	Productivity and education (*n* = 3)
Going shopping Washing dishes Washing clothes Taking out the rubbish Cooking Housekeeping (ironing, cleaning, making beds) Going to the dentist Driving Filling up on petrol Taking the car to the mechanic Going to the doctor Taking care of pets Paying bills Handling finances (investments, going to the bank) Going to the hairdresser Taking care of young children Taking care of ill people Using the computer Packing bags Using public transport Planning a trip Doing administrative tasks Sorting out cupboards, clothes Gardening/taking care of plants Surfing on the internet	Seeing sports in a sports stadium Shopping as leisure Board games Using social media Playing cards Doing crosswords, Sudoku Taking photos Home decorating Reading Visiting museums Going to the park Going to concerts Going to the theatre Going to the cinema Watching television Listening to music Listening to the radio Betting/gambling Going to the beach Going camping Going to the gym Playing videogames Having a nap Reading the newspaper	Going to the pool Playing team sports Going for a coffee Going for a walk Running Doing exercise Doing yoga, Pilates, tai chi Playing tennis/paddle tennis Hiking Riding a bicycle Travelling-tourism Going to parties Family events (weddings, communions) Talking on the phone Visiting friends/family members (ill) Visiting friends Eating at restaurants Dancing Taking the children to extracurricular activities Reading stories to children Spending time with friends Going out for a drink Organizing parties at home Going to funerals Going to shopping centers Visiting exhibitions Voting at elections	Working Job searching Studying

## Data Availability

If you need it, we could give you part of the data from the SPSS Statistics program, which is where we have all the data information.
